# Rice consumption and risk of cardiovascular disease: results from a pooled analysis of 3 U.S. cohorts^1^,^2^,^3^,^4^

**DOI:** 10.3945/ajcn.114.087551

**Published:** 2014-11-12

**Authors:** Isao Muraki, Hongyu Wu, Fumiaki Imamura, Francine Laden, Eric B Rimm, Frank B Hu, Walter C Willett, Qi Sun

**Affiliations:** 1 From the Departments of Nutrition (IM, HW, EBR, FBH, WCW, and QS), Epidemiology (FL, EBR, FBH, and WCW), and Environmental Health (FL), Harvard School of Public Health and Channing Division of Network Medicine (FL, EBR, FBH, WCW, and QS), Department of Medicine, Brigham and Women's Hospital and Harvard Medical School, Boston, MA, and the Medical Research Council Epidemiology Unit, Institute of Metabolic Science, University of Cambridge School of Clinical Medicine, Cambridge Biomedical Campus, Cambridge, United Kingdom (FI).

**Keywords:** cardiovascular disease, coronary artery disease, longitudinal study, rice, stroke

## Abstract

**Background:** Health concerns have been raised about rice consumption, which may significantly contribute to arsenic exposure. However, little is known regarding whether habitual rice consumption is associated with cardiovascular disease (CVD) risk.

**Objective:** We examined prospectively the association of white rice and brown rice consumption with CVD risk.

**Design:** We followed a total of 207,556 women and men [73,228 women from the Nurses’ Health Study (1984–2010), 92,158 women from the Nurses’ Health Study II (1991–2011), and 42,170 men from the Health Professionals Follow-Up Study (1986–2010)] who were free of CVD and cancer at baseline. Validated semiquantitative food-frequency questionnaires were used to assess consumption of white rice, brown rice, and other food items. Fatal and nonfatal CVD (coronary artery disease and stroke) was confirmed by medical records or self-reports.

**Results:** During 4,393,130 person-years of follow-up, 12,391 cases of CVD were identified. After adjustment for major CVD risk factors, including demographics, lifestyle, and other dietary intakes, rice consumption was not associated with CVD risk. The multivariable-adjuted HR of developing CVD comparing ≥5 servings/wk with <1 serving/wk was 0.98 (95% CI: 0.84, 1.14) for white rice, 1.01 (0.79, 1.28) for brown rice, and 0.99 (0.90, 1.08) for total rice. To minimize the potential impact of racial difference in rice consumption, we restricted the analyses to whites only and obtained similar results: the HRs of CVD for ≥5 servings/wk compared with <1 serving/wk were 1.04 (95% CI: 0.88, 1.22) for white rice and 1.01 (0.78, 1.31) for brown rice.

**Conclusions:** Greater habitual consumption of white rice or brown rice is not associated with CVD risk. These findings suggest that rice consumption may not pose a significant CVD risk among the U.S. population when consumed at current amounts. More prospective studies are needed to explore these associations in other populations.

## INTRODUCTION

Rice plays an important role as a staple food in more than half of the global populations, especially in the Asian population. Per capita rice consumption is also increasing in the United States (1). Meanwhile, rice consumption has been identified as an important route of arsenic exposure among populations not living in arsenic-endemic regions (2–4), as well as populations in arsenic-endemic regions, such as Bangladesh, Taiwan, and India, where groundwater is heavily contaminated by arsenic (5, 6). Recently, a health concern regarding rice consumption has been raised in the United States because rice grains, especially brown rice and its products, contain a high concentration of arsenic, according to a recent U.S. survey, and because in the National Health and Nutrition Examination Survey, urinary arsenic concentration was substantially higher among individuals who consumed rice than among those did not (7). Responding to this concern, the U.S. Food and Drug Administration reported that arsenic concentration in rice grains is too low to cause acute health effects of arsenic exposure, but the chronic effects of arsenic exposure from rice consumption have not been evaluated (8). Data regarding associations between rice consumption and risk of cardiovascular disease (CVD)^5^ are sparse and mixed. In a Japanese population who consumed white rice as a staple food, greater rice consumption was associated with lower mortality from CVD, especially coronary artery disease (CAD), in men, whereas in women, the association was not evident (9). In another Japanese study, there was a null association of risk of CVD, CAD, and stroke with rice consumption, although rice was the major source of arsenic intake in this Japanese population (10, 11). In contrast, among Chinese adults, greater carbohydrate intake mostly from white rice was associated with higher CAD incidence (12).

To our knowledge, no prospective study has been conducted to evaluate whether low rice consumption typical of Western populations is associated with CVD risk and whether white rice and brown rice intakes are differentially associated with CVD risk because of various contents of nutrients and arsenic, as well as different glycemic characteristics in these 2 types of rice. We therefore examined the prospective associations of white rice and brown rice with CVD risk among U.S. men and women participating in the Nurses’ Health Study (NHS), the Nurses’ Health Study II (NHSII), and the Health Professionals Follow-Up Study (HPFS).

## SUBJECTS AND METHODS

### Study population

The NHS was established in 1976 with a total enrollment of 121,701 female registered nurses (13). The NHSII, established in 1989, enrolled 116,430 younger nurses (14). The HPFS, established in 1986, consisted of 51,529 male health professionals (13). At baseline, to examine the associations between consumption of white rice or brown rice and the primary incidence of CVD, we excluded participants who reported a diagnosis of CVD (*n* = 3072 in 1984 for NHS, 1012 in 1991 for NHSII, and 4116 in 1986 for HPFS) and those who had missing data regarding white rice or brown rice consumption (*n* = 1014 for NHS, 812 for NHSII, and 1821 for HPFS). To minimize the impact of reverse causation caused by possible dietary changes after a diagnosis with chronic diseases, we excluded participants who reported a diagnosis of cancer (*n* = 4409 in 1984 for NHS, 1335 in 1991 for NHSII, and 2063 in 1986 for HPFS). We also excluded participants who had unusual amount of total energy intake (<500 or >3500 kcal/d for women and <800 or >4200 kcal/d for men), which meant unreliable response to food-frequency questionnaires (FFQs) (*n* = 2288 for NHSII and 1359 for HPFS). After these exclusions, 73,228 participants in NHS (1984–2010), 92,158 participants in NHSII (1991–2011), and 42,170 participants in HPFS (1986–2010) were included in the current analysis. The study protocols were approved by the institutional review boards of the Brigham and Women's Hospital and the Harvard School of Public Health. Completion and return of study questionnaires implied informed consent of the participants.

### Assessments of diet and other characteristics

In 1984, 1986, and every 4 y thereafter, semiquantitative FFQs with 118–166 items were mailed to the NHS participants to assess and update information on their usual intake of foods and beverages in the past year. The FFQs have been sent every 4 y to the NHSII participants since 1991 and to the HPFS participants since 1986. We asked the participants how often, on average, they consumed white rice and brown rice with a standard portion size of 1 cup (158 g for cooked white rice and 195 g for cooked brown rice). There were 9 possible responses, ranging from “never or less than once per month” to “6 or more times per day.” We used the same method to assess consumption of other foods. Nutrient intakes were estimated by multiplying the frequency of each food intake by the nutrient values for each food item with the specified serving size and summing the nutrient intake from all food items (15, 16). We derived an alternative Healthy Eating Index (aHEI) score as an indicator of adherence to healthy eating behavior by summarizing consumption of 11 foods and nutrients: vegetables, fruits, whole grains, sugar-sweetened beverages and fruit juice, nuts and legumes, red and processed meat, *trans* fat, long-chain n–3 fat, polyunsaturated fat, sodium, and alcohol (17). In the current study, we modified the aHEI score by excluding brown rice from the whole-grain consumption calculation. The validation study of the FFQ was previously evaluated by using multiple-day diet records as the reference method (see **Supplemental Methods**) (15, 16, 18, 19). The performance of our FFQ is similar to that of other FFQs (20–23). Based on our FFQ, consumption of white rice and brown rice contributed 15% and 7% of total arsenic intake on average, respectively (24).

In the follow-up questionnaires, we inquired about multiple demographic and lifestyle risk factors of CVD (see Supplemental Methods). In addition, we linked participants’ residential zip code with arsenic concentration in groundwater collected from county-specific data described in detail at the U.S. Geological Survey website (see Supplemental Methods) (25). We categorized the groundwater arsenic concentration into 3 groups based on the number of participants in each category and the U.S. Environmental Protection Agency regulation of arsenic concentration in drinking water: <3.0, 3.0–9.9, and ≥10.0 *μ*g/L (ppb) of groundwater arsenic concentration (26). Of note, we did not collect data on individual-level overall arsenic exposure such as urinary arsenic concentrations.

### Assessment of cardiovascular disease and death

The CVD outcomes included nonfatal myocardial infarction (MI), fatal CAD, and stroke (nonfatal or fatal). Briefly, the incidence of nonfatal MI and stroke was ascertained from the biennial follow-up questionnaires and confirmed by reviewing medical records with the World Health Organization criteria for MI (27) or the National Survey of Stroke criteria for stroke (see Supplemental Methods) (28). Deaths were identified by reports from next of kin or postal authorities or by searching the National Death Index. In a validation study among the NHS participants, 98% of deaths reported by kin or postal authorities were also identified by searching the National Death Index (29). In each cohort, the cause of death was confirmed by reviewing medical records or reliable sources such as autopsy records for more than 65% of deaths. Fatal cases of CAD and stroke were identified if CAD or stroke was listed as the cause of death in multiple sources, including autopsy reports, hospital records, and death certificates.

### Statistical analysis

For each participant, we calculated person-years from the date when the baseline questionnaire was returned to the date when participants were diagnosed with CVD, the date of death, or the end of follow-up (2010 for NHS and HPFS or 2011 for NHSII), whichever came first. To represent long-term dietary intake and minimize within-person variation, we calculated and used the cumulative average of intakes from all FFQs in our analyses (30). To minimize the impact of potential outliers and facilitate pooling the results from the 3 cohorts, we used the same cutoff points of rice consumption to categorize participants based on the considerations of consumption categories used in FFQs, distribution of rice consumption, and the hypothesis of interest that >2 servings/wk of rice consumption is associated with CVD risk. The categories used were: <1 serving/wk, 1 serving/wk, 2–4 servings/wk, and ≥5 servings/wk.

The HRs and 95% CIs of incident CVD were estimated for rice consumption by using time-dependent Cox proportional hazards regression after pooling data from 3 cohorts (31). The analysis was stratified jointly by age, cohorts, and calendar year and adjusted for various potential confounding factors, including baseline variables of sex, ethnicity, family history of MI, prevalent hypertension, hypercholesterolemia, or diabetes, and time-varying covariates of BMI, physical activity, cigarette smoking, alcohol intake, multivitamin use, menopausal status and postmenopausal hormone use (for women), oral contraceptive use (for NHSII only), current aspirin use, total energy intake, and the modified aHEI score. A test for linear trend was performed by modeling the median values for rice consumption categories as a continuous variable.

Because white rice consumption was largely different between Asians and other ethnicities, we also evaluated the associations among whites (*n* = 184,800) and Asians (*n* = 2660) separately. We examined potential interactions of consumption of white rice or brown rice with BMI, physical activity, smoking status, and the modified aHEI score by using a Wald test to evaluate the significance of the interaction terms between these variables and rice consumption. In addition, as an exploratory analysis, we analyzed the data stratified by groundwater arsenic concentration in the participant's county of residence instead of individual concentrations of overall arsenic exposure. To evaluate the robustness of our findings, we conducted 3 sensitivity analyses adjusting for individual dietary factors (including alcohol intake, polyunsaturated-to-saturated fat ratio, and intakes of *trans* fat, red meat, fish, fruits, vegetables, nuts, whole grains, coffee, and sugar-sweetened beverages) instead of the modified aHEI score, excluding participants who had prevalent hypertension at baseline or updating dietary information every 8 y instead of every 4 y (see Supplemental Methods). Statistical analyses were performed by using SAS 9.3 (SAS Institute). All *P* values were 2-sided, with statistical significance defined as *P* < 0.05.

## RESULTS

During 4,393,130 person-years of follow-up, 7719 participants developed CAD and 4672 participants developed stroke (NHS: 3060 CAD cases and 2703 stroke cases during 1,731,139 person-years; NHSII: 534 CAD cases and 494 stroke cases during 1,812,190 person-years; and HPFS: 4125 CAD cases and 1475 stroke cases during 849,801 person-years). At baseline, consumption of white rice and brown rice was inversely correlated with smoking, aspirin use, and oral contraceptive use (**Table 1**). Asians were more likely to consume white rice, but not brown rice, than other ethnicities. Greater white rice consumption was associated with lower consumption of whole grains and lower probability of having a family history of MI. Brown rice consumption was positively associated with physical activity, consumption of whole grains, and history of postmenopausal hormone use, as well as inversely correlated with BMI.

**TABLE 1:** Baseline characteristics of 73,228 women in the NHS (1984), 92,158 women in the NHSII (1991), and 42,170 men in the HPFS (1986) according to intake of white rice and brown rice^1^

	White rice intake, servings/wk				Brown rice intake, servings/wk			
	<1	1	2–4	≥5	<1	1	2–4	≥5
NHS								
*n*	48,473	18,852	5246	657	67,158	4552	1313	205
Age, y	50.6 ± 7.2	49.3 ± 7.1	49.3 ± 7.1	50.2 ± 6.9	50.1 ± 7.2	50.3 ± 7.1	50.9 ± 7.2	51.6 ± 6.6
BMI, kg/m^2^	25.0 ± 4.7	24.9 ± 4.7	24.8 ± 4.8	24.4 ± 4.7	25.0 ± 4.7	24.5 ± 4.4	24.2 ± 4.3	23.8 ± 3.9
Physical activity, MET-h/wk	13.9 ± 20.8	14.4 ± 21.4	15.1 ± 21.3	15.7 ± 26.4	13.6 ± 20.4	19.2 ± 25.7	21.8 ± 27.9	23.2 ± 32.2
Alcohol intake, g/d	6.6 ± 11.1	7.5 ± 11.3	8.1 ± 11.7	4.0 ± 8.2	6.8 ± 11.2	7.7 ± 11.2	6.6 ± 10.3	5.1 ± 8.5
Current smoker, %	24.6	23.9	21.3	16.3	24.6	20.0	15.8	11.2
Ethnicity, %								
White	98.8	98.1	93.6	50.7	97.8	98.0	96.5	90.7
Asian	0.2	0.2	1.7	42.0	0.7	0.4	1.1	2.9
African American	0.3	0.6	1.5	1.8	0.5	0.7	0.7	2.4
Hispanic/other	0.7	1.1	3.1	5.5	1.0	0.9	1.8	3.9
Family history of MI, %	39.1	38.6	37.6	30.6	38.9	39.0	35.7	37.1
Multivitamin use, %	37.3	36.0	37.4	37.3	36.0	46.0	52.5	48.8
Past or current PMH use, %	23.2	19.0	19.9	20.4	21.6	23.2	26.4	28.3
Hypertension, %	21.3	20.6	19.7	22.1	21.0	20.8	20.0	18.0
Hypercholesterolemia, %	8.0	7.6	8.5	9.0	7.8	9.1	11.6	8.8
Diabetes, %	2.9	2.6	2.8	4.6	2.9	2.6	2.9	2.9
Current aspirin use, %	66.3	68.7	67.4	55.9	67.3	64.7	58.5	50.2
Total energy intake, kcal/d	1668 ± 514	1854 ± 520	2025 ± 538	1986 ± 601	1733 ± 528	1837 ± 530	1949 ± 544	2001 ± 601
Glycemic load	98.6 ± 20.4	99.2 ± 17.6	102.0 ± 17.8	113.7 ± 21.2	98.8 ± 19.6	101.1 ± 18.6	106.9 ± 19.6	119.8 ± 24.2
White rice intake, servings/d	—	—	—	—	0.11 ± 0.15	0.10 ± 0.11	0.11 ± 0.17	0.11 ± 0.25
Brown rice intake, servings/d	0.03 ± 0.10	0.03 ± 0.07	0.03 ± 0.09	0.04 ± 0.15	—	—	—	—
Whole-grain intake,^2^ g/d	13.1 ± 12.7	12.2 ± 11.8	12.2 ± 12.5	9.3 ± 10.9	12.4 ± 12.1	15.6 ± 13.6	19.6 ± 16.0	25.1 ± 24.9
Modified aHEI score	45.6 ± 10.6	45.8 ± 9.6	47.1 ± 9.7	49.6 ± 10.1	45.2 ± 10.0	51.6 ± 10.2	55.3 ± 10.2	60.0 ± 10.8
NHSII								
*n*	51,041	27,100	12,002	2015	75,473	11,711	4327	647
Age, y	35.9 ± 4.7	36.3 ± 4.6	36.4 ± 4.6	36.3 ± 4.6	36.1 ± 4.7	36.0 ± 4.6	36.3 ± 4.5	36.6 ± 4.5
BMI, kg/m^2^	24.6 ± 5.4	24.6 ± 5.3	24.7 ± 5.3	24.1 ± 5.0	24.7 ± 5.4	24.1 ± 5.0	24.0 ± 4.9	24.1 ± 5.1
Physical activity, MET-h/wk	20.7 ± 27.6	20.5 ± 25.6	22.0 ± 28.3	21.2 ± 31.2	19.4 ± 25.6	26.0 ± 32.4	28.9 ± 31.4	38.4 ± 46.0
Alcohol intake, g/d	2.9 ± 6.0	3.3 ± 6.1	3.5 ± 6.3	2.3 ± 6.0	3.0 ± 6.0	3.7 ± 6.6	3.8 ± 6.2	3.1 ± 6.0
Current smoker, %	12.7	12.0	11.1	8.6	12.6	10.7	10.2	9.3
Ethnicity, %								
White	97.5	96.6	92.1	54.2	95.4	97.1	96.1	88.7
Asian	0.4	0.8	2.5	37.3	1.7	0.8	0.9	4.3
African American	0.9	1.3	3.0	4.3	1.4	1.1	1.7	2.8
Hispanic/other	1.2	1.3	2.5	4.3	1.5	0.9	1.2	4.2
Family history of MI, %	32.1	32.8	32.4	29.8	32.5	31.2	32.0	29.8
Multivitamin use, %	44.0	43.2	44.6	44.2	42.5	49.2	51.1	53.0
Past or current PMH use, %	3.2	3.0	3.0	2.3	3.2	2.7	2.7	3.7
Current OC use, %	11.3	10.3	9.5	8.6	10.9	10.7	8.7	7.9
Hypertension, %	6.4	6.1	6.2	6.6	6.5	5.4	5.7	3.9
Hypercholesterolemia, %	14.6	14.2	14.9	16.9	14.8	13.7	13.6	15.3
Diabetes, %	1.0	1.0	0.9	1.0	1.0	0.8	0.9	1.2
Current aspirin use, %	11.5	11.1	10.6	8.8	11.2	11.0	10.8	11.6
Total energy intake, kcal/d	1692 ± 531	1854 ± 529	2010 ± 546	2094 ± 600	1758 ± 543	1891 ± 536	2013 ± 548	2203 ± 583
Glycemic load	120.3 ± 22.4	120.6 ± 19.8	125.0 ± 19.9	136.4 ± 24.6	120.5 ± 21.6	122.9 ± 20.2	129.1 ± 21.1	140.9 ± 24.0
White rice intake, servings/d	—	—	—	—	0.15 ± 0.23	0.14 ± 0.14	0.17 ± 0.20	0.24 ± 0.35
Brown rice intake, servings/d	0.06 ± 0.12	0.06 ± 0.10	0.07 ± 0.14	0.08 ± 0.22	—	—	—	—
Whole-grain intake,^2^ g/d	17.2 ± 12.9	16.9 ± 12.2	17.2 ± 13.9	13.4 ± 12.6	16.3 ± 12.3	19.5 ± 13.8	22.1 ± 15.5	26.5 ± 19.3
Modified aHEI score	45.1 ± 10.4	46.1 ± 9.8	47.6 ± 9.8	49.3 ± 9.8	44.7 ± 9.9	49.7 ± 9.9	53.2 ± 10.0	56.4 ± 10.5
HPFS								
*n*	26,391	10,521	4400	858	34,257	5603	1970	340
Age, y	53.9 ± 9.6	51.4 ± 9.1	51.5 ± 9.2	51.4 ± 8.9	53.4 ± 9.5	51.1 ± 9.1	51.7 ± 9.4	52.0 ± 8.9
BMI, kg/m^2^	25.0 ± 5.0	25.0 ± 4.9	24.8 ± 5.1	24.3 ± 4.5	25.0 ± 4.9	24.8 ± 4.9	24.5 ± 5.2	24.0 ± 4.2
Physical activity, MET-h/wk	20.9 ± 29.1	21.9 ± 29.5	21.8 ± 31.2	21.0 ± 28.9	20.1 ± 28.5	25.2 ± 32.0	27.7 ± 32.9	34.1 ± 39.7
Alcohol intake, g/d	11.4 ± 15.7	11.6 ± 15.3	11.5 ± 14.9	8.5 ± 13.4	11.4 ± 15.6	11.6 ± 15.5	11.0 ± 14.1	8.2 ± 11.5
Current smoker, %	10.3	8.7	8.6	7.7	10.3	7.4	6.5	7.4
Ethnicity, %								
White	96.8	96.1	90.9	48.0	95.0	96.2	94.2	84.1
Asian	0.3	0.5	4.1	46.7	1.8	0.6	1.7	11.8
African American	2.2	2.4	2.5	3.3	2.3	2.3	2.4	2.9
Hispanic/other	0.7	1.0	2.6	2.0	1.0	0.9	1.7	1.2
Family history of MI, %	32.3	31.5	31.1	26.0	32.0	31.5	30.7	35.9
Multivitamin use	42.5	40.0	40.4	43.4	40.5	45.0	50.7	51.8
Hypertension, %	20.5	18.0	20.3	21.6	20.5	17.3	17.1	17.4
Hypercholesterolemia, %	10.2	10.4	11.8	11.9	10.2	11.2	12.8	15.0
Diabetes, %	2.5	2.3	2.5	3.4	2.6	2.1	2.2	2.4
Current aspirin use, %	27.0	26.6	25.9	19.0	26.8	25.9	25.2	21.2
Total energy intake, kcal/d	1911 ± 597	2097 ± 615	2243 ± 647	2189 ± 711	1963 ± 613	2115 ± 618	2214 ± 646	2327 ± 727
Glycemic load	122.0 ± 26.5	125.0 ± 23.5	129.9 ± 24.1	142.8 ± 28.1	122.4 ± 25.6	127.8 ± 24.8	135.3 ± 26.2	153.1 ± 29.4
White rice intake, servings/d	—	—	—	—	0.12 ± 0.22	0.13 ± 0.14	0.17 ± 0.20	0.24 ± 0.41
Brown rice intake, servings/d	0.06 ± 0.14	0.07 ± 0.11	0.09 ± 0.16	0.08 ± 0.24	—	—	—	—
Whole-grain intake,^2^ g/d	18.3 ± 17.4	17.6 ± 16.4	17.4 ± 18.0	14.0 ± 16.3	17.2 ± 16.4	20.2 ± 19.0	22.9 ± 20.6	29.5 ± 30.2
Modified aHEI score	49.7 ± 11.0	50.6 ± 10.5	51.2 ± 10.5	50.4 ± 10.2	49.1 ± 10.7	53.3 ± 10.2	56.2 ± 10.3	59.4 ± 9.6

1 Values are means ± SDs unless otherwise indicated. aHEI, alternate Healthy Eating Index; HPFS, Health Professionals Follow-Up Study; MET-h, metabolic equivalent task-hours; MI, myocardial infarction; NHS, Nurses’ Health Study; NHSII, Nurses’ Health Study II; OC, oral contraceptive; PMH, postmenopausal hormone.

2 Whole-grain intake was assessed from other than brown rice.

In the age-adjusted model, consumption of white rice, brown rice, and total rice was inversely associated with CVD risk (**Table 2**). After adjustment for demographic and lifestyle factors as well as modified aHEI score, these inverse associations were largely attenuated and no longer significant. Comparing extreme categories of rice consumption, the multivariable-adjusted HRs of CVD were 0.98 (95% CI: 0.84, 1.14) for white rice, 1.01 (0.79, 1.28) for brown rice, and 0.99 (0.90, 1.08) for total rice (*P*-trend = 0.69, 0.32, and 0.86, respectively). We did not detect statistically significant interactions of white rice and brown rice with BMI, physical activity, smoking status, and modified aHEI score in relation to CVD risk (**Supplemental Figure 1**).

**TABLE 2:** Prospective associations of rice consumption with cardiovascular disease among adults in the NHS, NHSII, and HPFS^1^

	Rice intake, servings/wk			
	<1	1	2–4	≥5	Every 3 servings/wk	*P*-trend
Cardiovascular disease						
White rice						
No. at risk	125,905	56,473	21,648	3530		
Cases/person-years	6175/1,979,490	3735/1,434,839	2261/878,523	220/100,278		
Model 1^2^	1.00	0.97 (0.93, 1.01)	0.96 (0.91, 1.01)	0.84 (0.73, 0.96)	0.94 (0.89, 0.98)	0.01
Model 2^3^	1.00	1.01 (0.97, 1.05)	1.00 (0.95, 1.05)	0.95 (0.82, 1.11)	0.99 (0.94, 1.05)	0.78
Model 3^4^	1.00	1.01 (0.97, 1.05)	1.02 (0.97, 1.07)	0.98 (0.84, 1.14)	1.01 (0.96, 1.06)	0.69
Brown rice						
No. at risk	176,888	21,866	7610	1192		
Cases/person-years	10,159/3,483,943	1436/582,370	727/296,734	69/30,083		
Model 1^2^	1.00	0.89 (0.84, 0.94)	0.85 (0.79, 0.92)	0.77 (0.61, 0.98)	0.82 (0.76, 0.88)	<0.001
Model 2^3^	1.00	0.96 (0.91, 1.02)	0.96 (0.89, 1.04)	0.86 (0.68, 1.10)	0.94 (0.87, 1.01)	0.10
Model 3^4^	1.00	1.01 (0.95, 1.07)	1.05 (0.97, 1.13)	1.01 (0.79, 1.28)	1.04 (0.96, 1.13)	0.32
Total rice						
No. at risk	88,619	61,433	41,849	15,655		
Cases/person-years	4104/1,222,338	3924/1,401,634	3738/1,467,592	625/301,565		
Model 1^2^	1.00	0.91 (0.87, 0.95)	0.87 (0.83, 0.91)	0.80 (0.73, 0.87)	0.88 (0.84, 0.91)	<0.001
Model 2^3^	1.00	0.98 (0.94, 1.02)	0.97 (0.92, 1.01)	0.92 (0.84, 1.00)	0.96 (0.92, 1.00)	0.06
Model 3^4^	1.00	1.00 (0.95, 1.04)	1.01 (0.96, 1.06)	0.99 (0.90, 1.08)	1.00 (0.96, 1.05)	0.86
Coronary artery disease						
White rice						
No. at risk	125,905	56,473	21,648	3530		
Cases/person-years	3848/1,979,490	2328/1,434,839	1417/878,523	126/100,278		
Model 1^2^	1.00	0.99 (0.94, 1.04)	0.98 (0.92, 1.04)	0.74 (0.62, 0.89)	0.93 (0.87, 0.98)	0.01
Model 2^3^	1.00	1.03 (0.98, 1.09)	1.03 (0.96, 1.09)	0.81 (0.66, 0.99)	0.98 (0.92, 1.05)	0.61
Model 3^4^	1.00	1.04 (0.98, 1.09)	1.05 (0.98, 1.12)	0.84 (0.69, 1.02)	1.01 (0.94, 1.07)	0.87
Brown rice						
No. at risk	176,888	21,866	7610	1192		
Cases/person-years	6328/3,483,943	899/582,370	457/296,734	35/30,083		
Model 1^2^	1.00	0.86 (0.80, 0.92)	0.83 (0.75, 0.91)	0.59 (0.42, 0.82)	0.76 (0.69, 0.84)	<0.001
Model 2^3^	1.00	0.94 (0.87, 1.01)	0.95 (0.86, 1.04)	0.66 (0.47, 0.92)	0.88 (0.80, 0.97)	0.01
Model 3^4^	1.00	0.99 (0.92, 1.06)	1.05 (0.95, 1.16)	0.80 (0.57, 1.12)	1.00 (0.91, 1.11)	0.95
Total rice						
No. at risk	88,619	61,433	41,849	15,655		
Cases/person-years	2547/1,222,338	2467/1,401,634	2309/1,467,592	396/301,565		
Model 1^2^	1.00	0.93 (0.88, 0.99)	0.86 (0.81, 0.91)	0.76 (0.69, 0.85)	0.86 (0.81, 0.90)	<0.001
Model 2^3^	1.00	1.01 (0.96, 1.07)	0.98 (0.92, 1.04)	0.88 (0.78, 0.98)	0.94 (0.90, 0.99)	0.03
Model 3^4^	1.00	1.03 (0.98, 1.09)	1.03 (0.97, 1.09)	0.97 (0.86, 1.08)	0.99 (0.94, 1.05)	0.81
Stroke						
White rice						
No. at risk	125,905	56,473	21,648	3530		
Cases/person-years	2327/1,979,490	1407/1,434,839	844/878,523	94/100,278		
Model 1^2^	1.00	0.94 (0.88, 1.01)	0.93 (0.86, 1.01)	1.02 (0.83, 1.26)	0.96 (0.89, 1.04)	0.31
Model 2^3^	1.00	0.97 (0.90, 1.03)	0.96 (0.89, 1.04)	1.23 (0.97, 1.55)	1.01 (0.93, 1.10)	0.85
Model 3^4^	1.00	0.97 (0.90, 1.03)	0.97 (0.89, 1.05)	1.25 (0.99, 1.57)	1.02 (0.94, 1.11)	0.67
Brown rice						
No. at risk	176,888	21,866	7610	1192		
Cases/person-years	3831/3,483,943	537/582,370	270/296,734	34/30,083		
Model 1^2^	1.00	0.94 (0.86, 1.03)	0.89 (0.79, 1.01)	1.14 (0.81, 1.59)	0.92 (0.82, 1.04)	0.20
Model 2^3^	1.00	1.00 (0.91, 1.10)	0.99 (0.87, 1.12)	1.28 (0.91, 1.80)	1.04 (0.92, 1.17)	0.58
Model 3^4^	1.00	1.03 (0.94, 1.14)	1.05 (0.92, 1.19)	1.39 (0.99, 1.96)	1.11 (0.98, 1.26)	0.12
Total rice						
No. at risk	88,619	61,433	41,849	15,655		
Cases/person-years	1557/1,222,338	1457/1,401,634	1429/1,467,592	229/301,565		
Model 1^2^	1.00	0.88 (0.82, 0.94)	0.88 (0.81, 0.94)	0.86 (0.75, 0.99)	0.92 (0.86, 0.98)	0.01
Model 2^3^	1.00	0.93 (0.86, 1.00)	0.95 (0.88, 1.03)	0.99 (0.86, 1.15)	0.99 (0.93, 1.06)	0.83
Model 3^4^	1.00	0.94 (0.87, 1.01)	0.98 (0.91, 1.06)	1.04 (0.89, 1.21)	1.02 (0.95, 1.10)	0.55

1 HPFS, Health Professionals Follow-Up Study; NHS, Nurses’ Health Study; NHSII, Nurses’ Health Study II.

2 HRs (95% CIs) in model 1 were estimated by Cox proportional hazards regression stratifying jointly by age (y), sex (male or female), and cohorts (NHS, NHSII, or HPFS).

3 HRs (95% CIs) in model 2 were estimated by Cox proportional hazards regression further adjusting for ethnicity (white, Asian, African American, and Hispanic/other), BMI (in kg/m^2^; <23.0, 23.0–24.9, 25.0–29.9, 30.0–34.9, or ≥35.0), smoking status [never smoked, past smoker, or currently smoke (1–14 or ≥15 cigarettes/d)], alcohol intake (0, 0.1–4.9, 5.0–9.9, 10.0–14.9, 15.0–29.9, or ≥30.0 g/d), physical activity (<3.0, 3.0–8.9, 9.0–17.9, 18.0–26.9, or ≥27.0 metabolic equivalent tasks × hours/wk), family history of myocardial infarction (yes or no), menopausal status and postmenopausal hormone use [premenopause, postmenopause (never, past, or current hormone use), for women], oral contraceptive use (never, past, or current use, for NHSII only), multivitamin use (yes or no), current aspirin use (yes or no), prevalent hypertension (yes or no), prevalent hypercholesterolemia (yes or no), prevalent diabetes (yes or no), and total energy intake (kcal/d).

4 HRs (95% CIs) in model 3 were estimated by Cox proportional hazards regression further adjusting for modified alternate Healthy Eating Index score (quintiles) as a summary measure of diet quality.

We did not observe positive associations of white rice, brown rice, and total rice with CAD risk either (Table 2). With adjustment for demographic, lifestyle, and dietary factors, comparing extreme categories, the HRs of CAD were 0.84 (95% CI: 0.69, 1.02) for white rice, 0.80 (0.57, 1.12) for brown rice, and 0.97 (0.86, 1.08) for total rice (*P*-trend = 0.87, 0.95, and 0.81, respectively). In terms of stroke risk, ≥5 servings/wk of white rice or brown rice was associated with a nonsignificant higher risk compared with <1 serving/wk. Such a positive trend was not found for total rice. The multivariable-adjusted HRs of stroke were 1.25 (95% CI: 0.99, 1.57; *P* = 0.06) for white rice, 1.39 (0.99, 1.96; *P* = 0.06) for brown rice, and 1.04 (0.89, 1.21; *P* = 0.64) for total rice (*P*-trend = 0.69, 0.12, and 0.55, respectively).

In a stratified analysis, the associations of white rice, brown rice, and total rice with CVD remained null among whites and Asians, respectively (see **Supplemental Table 1**). Comparing extreme categories, the multivariable-adjusted HRs of CVD for white rice were 1.04 (95% CI: 0.88, 1.22) among whites and 0.64 (0.30, 1.35) among Asians. The corresponding HRs were 1.01 (0.78, 1.31) and 0.53 (0.19, 1.45) for brown rice and 0.99 (0.90, 1.09) and 0.61 (0.24, 1.55) for total rice among whites and Asians, respectively.

To explore the potential interaction between rice consumption and background arsenic exposure in relation to CVD risk, we first examined the association of groundwater arsenic concentration in the participant's county of residence with CVD risk and found null associations: compared with <3.0 *μ*g/L (ppb) of groundwater arsenic concentration, the HRs of CVD risk were 0.95 (95% CI: 0.90, 1.01) for 3.0–9.9 *μ*g/L (ppb) and 1.01 (0.94, 1.08) for ≥10 *μ*g/L (ppb) (see **Supplemental Table 2**). The associations of rice consumption with CVD risk, however, appeared to be somewhat modified by groundwater arsenic concentration (see **Supplemental Table 3**) (*P*-interaction = 0.05 for white rice, 0.95 for brown rice, and 0.14 for total rice). Among participants living in low arsenic areas [<3.0 *μ*g/L (ppb) of groundwater arsenic concentration], white rice consumption was positively associated with CVD risk, whereas among those who lived in modest or high arsenic areas [3.0–9.9 and ≥10.0 *μ*g/L (ppb)], no association was found. However, the HR for each consumption amount was not significant probably because of limited statistical power in the stratified analyses. Regarding risk of CAD or stroke, interactions of total rice consumption and groundwater arsenic concentration were not found (see **Supplemental Table 4**).

In sensitivity analyses (adjusting for individual dietary factors instead of modified aHEI score, excluding participants who had prevalent hypertension at baseline, and updating dietary information every 8 y instead of every 4 y), the associations of CVD were largely similar to the results from primary analyses (see **Supplemental Table 5**).

## DISCUSSION

In these well-characterized large cohorts of U.S. male and female health professionals, we did not find significant associations between rice consumption and risk of developing CVD or CAD independently of demographic, lifestyle, and dietary risk factors of CVD. These null associations were largely similar between whites and Asians.

Rice consumption is known to contribute to arsenic exposure among populations who live in arsenic-endemic regions in Bangladesh, Taiwan, and India (5, 6). In the United States, rice and rice products are also one of the major dietary sources of exposure to total and inorganic arsenic (24, 32). Chronic exposure to arsenic, especially inorganic arsenic, may be atherogenic through multifaceted detrimental effects on blood pressure, systemic inflammation, oxidative stress, and endothelial dysfunction (33). In arsenic-endemic regions, high arsenic concentration in drinking water was associated with increased risk of CVD (34–37). However, findings from non–arsenic-endemic areas were mixed. In 3 ecological studies, regional arsenic concentration in groundwater was associated with an increased CVD risk in Spain and the United States (38–40), although in other 2 ecological studies in the United States, such a positive association was not found (41, 42). In the only prospective study, higher urinary concentration of inorganic plus methylated organic arsenic species (arsenite, arsenate, monomethylarsenate, and dimethylarsinate; median concentration of 9.7 *μ*g/g creatinine with a range of 0.1–183.4 *μ*g/g creatinine) was associated with elevated risks of CVD, CAD, and stroke among U.S. adults living in Arizona, Oklahoma, and the Dakotas, independently of age, sex, educational levels, smoking status, BMI, and plasma concentration of LDL cholesterol (43).

In contrast to the evidence regarding arsenic concentration in drinking water, evidence on the association of rice consumption as a route of arsenic exposure in relation to CVD risk is sparse. The current analysis provides new evidence suggesting that in U.S. populations with overall low rice intake, rice consumption is unlikely to contribute to an elevated risk of CVD or CAD. Our findings are in line with the findings in a Japanese population who, on average, consumed a much greater amount of white rice than did our population (9, 10). Meanwhile, in the current analysis, consumption of 5 or more servings/wk of white rice or brown rice was nonsignificantly associated with a higher stroke risk. However, such an association was largely abolished when we examined the same amount of total rice intake in relation to stroke risk. Also, in the exploratory analysis stratifying by the groundwater arsenic concentrations in the participant's county of residence, we found a marginal interaction between rice consumption and groundwater arsenic concentration in the participant's county of residence in relation to CVD risk: white rice consumption was positively associated with CVD risk only in regions where groundwater arsenic concentration was low. These nonsignificant findings, however, can be detected simply by chance, and further investigations with individual-level data of arsenic exposure from dietary and environmental routes are warranted.

The possible reasons for the lack of positive associations between rice consumption and CVD risk are worth discussing. First, in a study conducted among U.S. pregnant women, rice consumption explained only 4% of the variability of urinary total arsenic concentration, whereas arsenic intake from drinking water explained 12% (2). Because of the relatively low contribution of rice consumption to arsenic exposure, potential health effects of arsenic exposure from rice consumption may be easily masked by those of arsenic exposure from other routes such as drinking water. Second, arsenic concentrations in rice grains may vary substantially across rice cultivars, cultivating methods (flooding or nonflooding), irrigation water usage, and arsenic contents in soil and irrigation water (44–46). Moreover, cooking methods and arsenic contents in cooking water may modify arsenic contents in cooked rice (46). Furthermore, the bioavailability of arsenic in rice also varies across rice cultivars and cooking methods (47, 48). The variation of bioavailable arsenic concentrations in cooked rice may dilute the importance of arsenic exposure from rice consumption. Last, whole rice grains (brown rice) contain insoluble fiber, magnesium, vitamin E (49), and phytochemicals (50, 51) that may jointly have beneficial effects on cardiovascular health (52–55) through lowering blood pressure levels (56–58) and blood cholesterol concentration (50, 58–60), improving glucose metabolism (61), and reducing oxidative stress (62). These beneficial effects of whole-grain rice may counteract the adverse effects exerted by arsenic exposure from rice consumption. In contrast, refined rice grains (white rice) contain less arsenic and the abovementioned nutrients because rice bran rich in these substances is removed during polishing (63). The polishing process may also make rice grains easily absorbable and leads to an increased glycemic index and glycemic load, which is a dietary risk factor of CVD (64). However, in our populations, white rice was only a minor contributor to the overall dietary glycemic index or load.

Strengths of the current study include a prospective study design, large sample size, and repeated measurements of exposure and various confounders. The current study also has several limitations. First, rice consumption in the United States was much lower than that in Asian countries, and we therefore are unable to extrapolate whether at much higher intakes, rice intake is associated with CVD risk from the current findings. Second, our study participants primarily consisted of health professionals with European ancestry, further limiting the generalizability of our findings to populations of different ethnicities. Third, measurement error in assessments of rice consumption is inevitable, and we have incomplete knowledge of the extent to which such error may bias our results. To minimize random errors, we calculated and used the cumulative average of rice consumption during follow-up. Because of the prospective study design, measurement errors of rice consumption are more likely to be random and thus bias the associations toward the null. Fourth, we had no individual data on water usage from public water supplies, private wells, bottled water, and other sources. The county-level groundwater arsenic concentration may not necessarily reflect the actual arsenic exposure from drinking water. Because the measurement error is unlikely to be related with disease outcomes, such nondifferential errors will be more likely to dilute true associations to the null. Last, we cannot eliminate the possibility that our findings were due to chance or residual confounding. For example, serum concentrations of LDL cholesterol were not available in all 3 cohorts. Although we adjusted for a self-reported hypercholesterolemia, which was reliable to use as a covariate (65), some residual confounding may still exist (66).

In conclusion, greater consumption of white rice or brown rice was not associated with an increased risk of CVD or CAD in U.S. men and women. Although a recent report from *Consumer Reports* magazine recommended limiting rice consumption to 2 servings/wk or less (7), the current evidence does not lend support to such a recommendation. Further evidence is nevertheless needed to elucidate the interrelationships among arsenic exposures from multiple sources, intake of various types of rice grains, and CVD risk, as well as other disease outcomes.

## Supplementary Material

Supplemental data

## Footnotes

5 Abbreviations used: aHEI, alternative Healthy Eating Index; CAD, coronary artery disease; CVD, cardiovascular disease; FFQ, food-frequency questionnaire; HPFS, Health Professionals Follow-Up Study; MI, myocardial infarction; NHS, Nurses’ Health Study; NHSII, Nurses’ Health Study II.
