# Supplementary material for: Rice consumption and risk of cardiovascular disease: results from a pooled analysis of 3 U.S. cohorts1
Source: Am J Clin Nutr. 2014 Nov 12;101(1):164–72. doi: 10.3945/ajcn.114.087551 (PMC4266886; doi:10.3945/ajcn.114.087551)
Supplement: Supplemental data [file supp_101_1_164__index.html]

Supplemental data 

# Rice consumption and risk of cardiovascular disease: results from a pooled analysis of 3 U.S. cohorts

## Supplemental data

**Files in this Data Supplement:**

- Supplemental data - Methods, Tables 1-5, and Figure 1
