# Supplementary material for: Rice consumption and risk of cardiovascular disease: results from a pooled analysis of 3 U.S. cohorts1
Source: Am J Clin Nutr. 2014 Nov 12;101(1):164–72. doi: 10.3945/ajcn.114.087551 (PMC4266886; doi:10.3945/ajcn.114.087551)
Supplement: Supplemental data [file 114.087551_ajcn087551SupplementaryData1.docx]

**Online Supplementary Material**

Muraki I, Wu H, Imamura F, et al. Rice consumption and risk of cardiovascular disease: Results from a pooled data analysis of three U.S. cohorts

**Supplementary Methods.**

**Supplementary Table 1.** Multivariable-adjusted association of rice consumption with cardiovascular disease among Caucasians and Asians in NHS, NHSII, and HPFS

**Supplementary Table 2.** Prospective association of groundwater arsenic levels with cardiovascular disease, coronary heart disease, and stroke among adults in NHS, NHSII, and HPFS

**Supplementary Table 3.** Stratified by groundwater arsenic levels, multivariable-adjusted association of rice consumption with cardiovascular disease among adults in NHS, NHSII, and HPFS

**Supplementary Table 4.** Stratified by groundwater arsenic levels, multivariable-adjusted association of total rice consumption with coronary heart disease and stroke among adults in NHS, NHSII, and HPFS

**Supplementary Table 5.** Results from sensitivity analyses, multivariable-adjusted hazard ratios (95% confidence intervals) of cardiovascular disease for ≥5 servings/week of rice consumption compared with <1 serving/week

**Supplementary Figure 1.** Multivariable-adjusted hazard ratios of cardiovascular disease according to combined categories of white rice consumption or brown rice consumption with body mass index, physical activity, smoking status, and modified alternate Healthy Eating Index score, respectively

**Reference.**

**Supplementary Methods.**

**Validation of food frequency questionnaire**

The validity of the food frequency questionnaire (FFQ) was examined with dietary records used as the reference among 173 Nurses’ Health Study (NHS) participants in 1980 and 127 Health Professionals Follow-up Study (HPFS) participants in 1986 (1–4). The correlation coefficients corrected for within-person variation between estimates derived from FFQ and dietary records were 0.53 for white rice, and 0.41 for brown rice in men (4). The modest correlations may be due to the relative infrequent consumption of white and brown rice during the 14 days of diet recording from the validation studies.

**Assessment of covariates**

In the biennial follow-up questionnaires, we inquired about and updated information on cardiovascular risk factors, such as body weight and height, cigarette smoking, physical activity, multivitamin use, family history of myocardial infarction (MI), current aspirin use, prevalent hypertension, prevalent hypercholesterolemia, prevalent diabetes, and other factors. Among women, we ascertained menopausal status, postmenopausal hormone use, and oral contraceptive use (NHSII only). Body mass index was calculated as body weight (kg) divided by height squared (m^2^). Physical activity was estimated as a total of metabolic equivalent of task (MET) for each activity multiplied by time spend for each activity during a week (MET-hours/week). One MET was equal to the resting metabolic rate which is energy expenditure during quiet seating, and MET score was calculated as the ratio of the metabolic rate during a specific activity to the resting metabolic rate. Current aspirin use was defined as taking at least one aspirin tablet per week.

**Data of groundwater arsenic levels**

The data of arsenic levels in groundwater samples from each county were obtained at the U.S. Geological Survey website (5). We used the 75 percentile of arsenic levels in groundwater samples from each county as a groundwater arsenic level of each county which was used to make the U.S. map of arsenic levels in groundwater (5). Then, we linked the groundwater arsenic level of each county with individual data from all cohorts using their geocode which was coded based on their address in 1986 for NHS and HPFS, and in 1989 for NHSII. Excluding participants who had missing data on groundwater arsenic levels due to no available data for their county or because they were living outside the continental U.S., we included 47,646 women from NHS, 50,200 women from NHSII, and 26,315 men from HPFS in the stratified analysis (65.1% of NHS participants, 54.5% of NHSII participants, and 62.4% of HPFS participants included in the main analysis).

**Diagnosis criteria of cardiovascular disease**

Incidence of non-fatal MI and stroke was ascertained from the biennial follow-up questionnaires. If a participant reported a diagnosis and hospitalization for MI or stroke, we first obtained consent from the participant by letter or by phone to obtain and review her or his medical records. Study physicians blinded to exposure status then reviewed medical records and confirm/refute self-reported diagnosis according to findings of blood tests, electrocardiography, and neuroimaging (computerized tomography/ magnetic resonance imaging). To confirm non-fatal MI, we used the World Health Organization criteria which require typical symptoms plus either diagnostic electrocardiographic findings or elevated levels of blood cardiac enzyme (6). To confirm non-fatal stroke, we used the criteria defined in the National Survey of Stroke which require a sudden- or rapid-onset neurologic deficit persisting for 24 hours or until death (7).

Deaths were identified by reports from next of kin or postal authorities, or by searching the National Death Index. Fatal coronary heart disease (CHD) and stroke cases were identified if they were listed as the cause of death in multiple sources including autopsy reports, hospital records, and death certificates. Fatal cardiovascular disease (CVD) cases were then confirmed if CHD or stroke had been recorded without any other more apparent or plausible cause of death. In a validation study among the NHS participants, 98% of deaths reported by kin or postal authorities was also identified by searching the National Death Index (8).

CVD cases were considered probable if study participants confirmed diagnoses in telephone interviews or through mail, but medical records were not obtained [1,945 of 5,763 (34%) in NHS, 485 of 1,028 (47%) in NHSII, and 1,208 of 5,600 (22%) in HPFS]. In the current analysis, we included both confirmed and probable CVD cases.

**Sensitivity analysis**

To examine the robustness of our findings, we conducted three sensitivity analyses: 1) we adjusted for individual lifestyle habits and dietary factors instead of modified alternate Healthy Eating Index score to assess the potential impact of residual confounding caused by summarizing multiple dietary factors, 2) we excluded participants who had a history of hypertension at baseline to minimize the impact of hypertension on the associations of interest; and 3) we updated dietary information every eight years instead of every four years to minimize the impact of potential reverse causality caused by dietary change immediately before the incidence of CVD.

**Supplementary Table 1.** Multivariable-adjusted association of rice consumption with cardiovascular disease among Caucasians and Asians in NHS, NHSII, and HPFS

|  | Rice consumption levels, serving/week | | | | Every  3 servings/week | P value  for trend |
| --- | --- | --- | --- | --- | --- | --- |
|  | <1 | 1 | 2-4 | ≥5 |  |  |
| White rice |  |  |  |  |  |  |
| Caucasians |  |  |  |  |  |  |
| No. at risk | 123,208 | 54,780 | 19,962 | 1,837 |  |  |
| Case/person-years | 6,039/1,939,921 | 3,659/1,401,749 | 2,164/832,429 | 145/57,911 |  |  |
| Adjusted HR (95% CI)^1^ | 1.00 | 1.01 (0.97, 1.05) | 1.02 (0.97, 1.07) | 1.04 (0.88, 1.22) | 1.02 (0.97, 1.08) | 0.44 |
| Asians |  |  |  |  |  |  |
| No. at risk | 362 | 303 | 567 | 1,428 |  |  |
| Case/person-years | 11/4,375 | 8/5,157 | 26/11,471 | 58/34,519 |  |  |
| Adjusted HR (95% CI)^1^ | 1.00 | 0.77 (0.27, 2.16) | 1.10 (0.50, 2.44) | 0.64 (0.30, 1.35) | 0.76 (0.54, 1.07) | 0.11 |
| Brown rice |  |  |  |  |  |  |
| Caucasians |  |  |  |  |  |  |
| No. at risk | 170,231 | 21,228 | 7,282 | 1,046 |  |  |
| Case/person-years | 9,856/3,354,974 | 1,394/566,063 | 697/284,564 | 60/26,430 |  |  |
| Adjusted HR (95% CI)^1^ | 1.00 | 1.00 (0.94, 1.06) | 1.04 (0.96, 1.12) | 1.01 (0.78, 1.31) | 1.03 (0.95, 1.12) | 0.43 |
| Asians |  |  |  |  |  |  |
| No. at risk | 2,356 | 142 | 88 | 74 |  |  |
| Case/person-years | 82/45,987 | 6/3,877 | 10/3,464 | 5/2,194 |  |  |
| Adjusted HR (95% CI)^1^ | 1.00 | 1.31 (0.51, 3.37) | 1.59 (0.74, 3.44) | 0.53 (0.19, 1.45) | 0.84 (0.51, 1.38) | 0.50 |
| Total rice |  |  |  |  |  |  |
| Caucasians |  |  |  |  |  |  |
| No. at risk | 86,818 | 59,788 | 39,907 | 13,274 |  |  |
| Case/person-years | 4,020/1,199,071 | 3,843/1,371,240 | 3,627/1,415,004 | 517/246,717 |  |  |
| Adjusted HR (95% CI)^1^ | 1.00 | 0.99 (0.95, 1.04) | 1.01 (0.96, 1.06) | 0.99 (0.90, 1.09) | 1.00 (0.96, 1.05) | 0.88 |
| Asians |  |  |  |  |  |  |
| No. at risk | 221 | 280 | 531 | 1,628 |  |  |
| Case/person-years | 7/2,436 | 6/4,221 | 21/10,672 | 69/38,195 |  |  |
| Adjusted HR (95% CI)^1^ | 1.00 | 0.69 (0.19, 2.53) | 0.94 (0.34, 2.57) | 0.61 (0.24, 1.55) | 0.76 (0.52, 1.13) | 0.17 |

^1^ HRs (95% CIs) were estimated by Cox proportional hazard regression stratifying jointly by age (years), gender (male or female), and cohorts (NHS, NHSII, or HPFS), and adjusting for body mass index (<23.0, 23.0-24.9, 25.0-29.9, 30.0-34.9, or ≥35.0 kg/m^2^), smoking status [never smoked, past smoker, currently smoke (1-14, or ≥15 cigarettes/day)], physical activity (<3.0, 3.0-8.9, 9.0-17.9, 18.0-26.9, or ≥27.0 metabolic equivalent of tasks × hours/week), family history of myocardial infarction (yes or no), menopausal status and postmenopausal hormone use [pre-menopause, post-menopause (never, past, or current hormone use)], oral contraceptive use (never, past, or current use, for NHSII only), multivitamin use (yes or no), current aspirin use (yes or no), prevalent hypertension (yes or no), prevalent hypercholesterolemia (yes or no), prevalent diabetes (yes or no), total energy intake (kcal/day), and modified alternate Healthy Eating Index score (quintiles).

HPFS, Health Professionals Follow-up Study; NHS, Nurses’ Health Study.

**Supplementary Table 2.** Prospective association of groundwater arsenic levels with cardiovascular disease, coronary heart disease, and stroke among adults in NHS, NHSII, and HPFS

|  | Arsenic levels in groundwater of resident area, μg/L (ppb) | | |
| --- | --- | --- | --- |
|  | <3 | 3.0-9.9 | 10.0+ |
| No at risk | 80,220 | 26,425 | 17,516 |
| Person-years | 1,718,742 | 559,505 | 369,278 |
| Cardiovascular disease |  |  |  |
| No of cases | 5,080 | 1,537 | 1,056 |
| Mode11^1^ | 1.00 | 0.94 (0.89, 1.00) | 0.97 (0.91, 1.04) |
| Model2^2^ | 1.00 | 0.96 (0.90, 1.01) | 1.00 (0.93, 1.06) |
| Model3^3^ | 1.00 | 0.95 (0.90, 1.01) | 1.01 (0.94, 1.08) |
| Coronary heart disease |  |  |  |
| No of cases | 3,169 | 966 | 646 |
| Mode11^1^ | 1.00 | 0.93 (0.86, 1.00) | 0.94 (0.87, 1.03) |
| Model2^2^ | 1.00 | 0.95 (0.88, 1.02) | 0.97 (0.89, 1.05) |
| Model3^3^ | 1.00 | 0.94 (0.88, 1.01) | 0.98 (0.90, 1.07) |
| Stroke |  |  |  |
| No of cases | 1,911 | 571 | 410 |
| Mode11^1^ | 1.00 | 0.97 (0.88, 1.06) | 1.03 (0.92, 1.14) |
| Model2^2^ | 1.00 | 0.97 (0.88, 1.07) | 1.05 (0.94, 1.16) |
| Model3^3^ | 1.00 | 0.97 (0.89, 1.07) | 1.05 (0.95, 1.17) |

^1^ HRs (95% CIs) in model 1 were estimated by Cox proportional hazard regression stratifying by age (years), gender (male or female), and cohorts (NHS, NHSII, or HPFS).

^2^ HRs (95% CIs) in model 2 were estimated by Cox proportional hazard regression further adjusting for ethnicity (Caucasian, Asian, African American, and Hispanic/others), body mass index (<23.0, 23.0-24.9, 25.0-29.9, 30.0-34.9, or ≥35.0 kg/m^2^), smoking status [never smoked, past smoker, currently smoke (1-14, or ≥15 cigarettes/day)], alcohol intake (0, 0.1-4.9, 5.0-9.9, 10.0-14.9, 15.0-29.9, or ≥30.0 g/day), physical activity (<3.0, 3.0-8.9, 9.0-17.9, 18.0-26.9, or ≥27.0 metabolic equivalent of tasks × hours/week), family history of myocardial infarction (yes or no), menopausal status and postmenopausal hormone use [pre-menopause, post-menopause (never, past, or current hormone use), for women], oral contraceptive use (never, past, or current use, for NHSII only), multivitamin use (yes or no), current aspirin use (yes or no), prevalent hypertension (yes or no), prevalent hypercholesterolemia (yes or no), prevalent diabetes (yes or no), and total energy intake (kcal/day).

^3^ HRs (95% CIs) in model 3 were estimated by Cox proportional hazard regression further adjusting for modified alternate Healthy Eating Index score (quintiles).

HPFS, Health Professionals Follow-up Study; NHS, Nurses’ Health Study.

**Supplementary Table 3.** Stratified by groundwater arsenic levels, multivariable-adjusted association of rice consumption with cardiovascular disease among adults in NHS, NHSII, and HPFS

|  | Rice intake levels, serving/week | | | | Every  3 servings/week | *P* value  for trend |
| --- | --- | --- | --- | --- | --- | --- |
|  | <1 | 1 | 2-4 | ≥5 |  |  |
| Groundwater arsenic levels <3.0 μg/L (ppb) |  |  |  |  |  |  |
| White rice |  |  |  |  |  |  |
| No at risk | 20,248 | 9,037 | 3,076 | 344 |  |  |
| Case/person-years | 2,457/771,742 | 1,579/571,118 | 961/344,497 | 83/31,386 |  |  |
| Adjusted HR (95% CI)^1^ | 1.00 | 1.04 (0.98, 1.11) | 1.06 (0.98, 1.15) | 1.23 (0.97, 1.56) | 1.08 (1.00, 1.18) | 0.05 |
| Brown rice |  |  |  |  |  |  |
| No at risk | 28,344 | 3,231 | 982 | 148 |  |  |
| Case/person-years | 4,154/1,367,731 | 602/226,807 | 293/113,431 | 31/10,774 |  |  |
| Adjusted HR (95% CI)^1^ | 1.00 | 1.03 (0.94, 1.13) | 1.06 (0.94, 1.20) | 1.14 (0.79, 1.63) | 1.08 (0.95, 1.22) | 0.25 |
| Total rice |  |  |  |  |  |  |
| No at risk | 14,863 | 9,786 | 6,099 | 1,957 |  |  |
| Case/person-years | 1,624/477,651 | 1,614/557,114 | 1,592/577,774 | 250/106,202 |  |  |
| Adjusted HR (95% CI)^1^ | 1.00 | 1.01 (0.94, 1.08) | 1.06 (0.99, 1.14) | 1.09 (0.95, 1.26) | 1.07 (1.00, 1.14) | 0.06 |
| Groundwater arsenic levels 3.0-9.9 μg/L (ppb) |  |  |  |  |  |  |
| White rice |  |  |  |  |  |  |
| No at risk | 28,594 | 13,138 | 5,071 | 712 |  |  |
| Case/person-years | 790/256,713 | 468/180,743 | 253/109,745 | 26/12,304 |  |  |
| Adjusted HR (95% CI)^1^ | 1.00 | 1.03 (0.91, 1.16) | 0.96 (0.83, 1.12) | 1.01 (0.65, 1.58) | 0.96 (0.83, 1.13) | 0.65 |
| Brown rice |  |  |  |  |  |  |
| No at risk | 40,354 | 5,058 | 1,837 | 266 |  |  |
| Case/person-years | 1,272/440,587 | 171/75,522 | 84/39,350 | 10/4,048 |  |  |
| Adjusted HR (95% CI)^1^ | 1.00 | 0.93 (0.79, 1.10) | 0.89 (0.71, 1.12) | 1.38 (0.73, 2.59) | 0.95 (0.75, 1.20) | 0.66 |
| Total rice |  |  |  |  |  |  |
| No at risk | 19,931 | 14,287 | 9,751 | 3,546 |  |  |
| Case/person-years | 512/153,878 | 526/182,261 | 425/183,965 | 74/39,403 |  |  |
| Adjusted HR (95% CI)^1^ | 1.00 | 1.03 (0.91, 1.17) | 0.93 (0.81, 1.07) | 0.92 (0.71, 1.21) | 0.92 (0.81, 1.04) | 0.18 |
| Groundwater arsenic levels ≥10.0 μg/L (ppb) |  |  |  |  |  |  |
| White rice |  |  |  |  |  |  |
| No at risk | 8,623 | 3,735 | 1,424 | 223 |  |  |
| Case/person-years | 554/158,578 | 291/115,519 | 181/80,063 | 30/15,118 |  |  |
| Adjusted HR (95% CI)^1^ | 1.00 | 0.88 (0.76, 1.02) | 0.83 (0.69, 0.99) | 0.81 (0.51, 1.30) | 0.84 (0.70, 1.00) | 0.06 |
| Brown rice |  |  |  |  |  |  |
| No at risk | 11,764 | 1,572 | 584 | 85 |  |  |
| Case/person-years | 848/286,526 | 121/50,642 | 77/28,652 | 10/3,458 |  |  |
| Adjusted HR (95% CI)^1^ | 1.00 | 0.88 (0.72, 1.07) | 1.01 (0.79, 1.29) | 1.08 (0.56, 2.05) | 1.01 (0.79, 1.29) | 0.92 |
| Total rice |  |  |  |  |  |  |
| No at risk | 5,809 | 4,260 | 2,840 | 1,096 |  |  |
| Case/person-years | 353/96,094 | 321/110,148 | 309/128,039 | 73/34,998 |  |  |
| Adjusted HR (95% CI)^1^ | 1.00 | 0.98 (0.84, 1.14) | 0.87 (0.74, 1.03) | 0.91 (0.68, 1.22) | 0.90 (0.78, 1.04) | 0.14 |

^1^ HRs (95% CIs) were estimated by Cox proportional hazard regression stratifying by age (years), gender (male or female), and cohorts (NHS, NHSII, or HPFS), and adjusting for ethnicity (Caucasian, Asian, African American and Hispanic/others), body mass index (<23.0, 23.0-24.9, 25.0-29.9, 30.0-34.9, or ≥35.0 kg/m^2^), smoking status [never smoked, past smoker, currently smoke (1-14, or ≥15 cigarettes/day)], physical activity (<3.0, 3.0-8.9, 9.0-17.9, 18.0-26.9, or ≥27.0 metabolic equivalent of tasks × hours/week), family history of myocardial infarction (yes or no), menopausal status and postmenopausal hormone use [pre-menopause, post-menopause (never, past, or current hormone use)], oral contraceptive use (never, past, or current use, for NHSII only), multivitamin use (yes or no), current aspirin use (yes or no), prevalent hypertension (yes or no), prevalent hypercholesterolemia (yes or no), prevalent diabetes (yes or no), total energy intake (kcal/day), and modified alternate Healthy Eating Index score (quintiles).

P value for interaction was 0.05 for white rice, 0.95 for brown rice, and 0.14 for total rice.

HPFS, Health Professionals Follow-up Study; NHS, Nurses’ Health Study.

**Supplementary Table 4.** Stratified by groundwater arsenic levels, multivariable-adjusted association of total rice consumption with coronary heart disease and stroke among adults in NHS, NHSII, and HPFS

|  | Total rice intake levels, serving/week | | | | Every  3 servings/week | P value  for trend |
| --- | --- | --- | --- | --- | --- | --- |
|  | <1 | 1 | 2-4 | ≥5 |  |  |
| Coronary heart disease |  |  |  |  |  |  |
| Groundwater arsenic levels <3.0 μg/L (ppb) |  |  |  |  |  |  |
| No at risk | 14,863 | 9,786 | 6,099 | 1,957 |  |  |
| Cases/person-years | 1,003/477,651 | 1,007/557,114 | 1,000/577,774 | 159/106,202 |  |  |
| Adjusted HR (95% CI)^1^ | 1.00 | 1.04 (0.95, 1.14) | 1.10 (1.00, 1.20) | 1.05 (0.88, 1.25) | 1.06 (0.98, 1.15) | 0.16 |
| Groundwater arsenic levels 3.0-9.9 μg/L (ppb) |  |  |  |  |  |  |
| No at risk | 19,931 | 14,287 | 9,751 | 3,546 |  |  |
| Cases/person-years | 329/153,878 | 340/182,261 | 248/183,965 | 49/39,403 |  |  |
| Adjusted HR (95% CI)^1^ | 1.00 | 1.06 (0.91, 1.24) | 0.87 (0.73, 1.04) | 1.03 (0.74, 1.42) | 0.90 (0.77, 1.05) | 0.20 |
| Groundwater arsenic levels ≥10.0 μg/L (ppb) |  |  |  |  |  |  |
| No at risk | 5,809 | 4,260 | 2,840 | 1,096 |  |  |
| Cases/person-years | 214/96,094 | 202/110,148 | 184/128,039 | 46/34,998 |  |  |
| Adjusted HR (95% CI)^1^ | 1.00 | 1.04 (0.85, 1.27) | 0.87 (0.70, 1.07) | 0.89 (0.62, 1.28) | 0.87 (0.73, 1.05) | 0.14 |
| Stroke |  |  |  |  |  |  |
| Groundwater arsenic levels <3.0 μg/L (ppb) |  |  |  |  |  |  |
| No at risk | 14,863 | 9,786 | 6,099 | 1,957 |  |  |
| Cases/person-years | 621/477,651 | 607/557,114 | 592/577,774 | 91/106,202 |  |  |
| Adjusted HR (95% CI)^1^ | 1.00 | 0.96 (0.86, 1.08) | 1.01 (0.90, 1.14) | 1.18 (0.94, 1.50) | 1.08 (0.96, 1.20) | 0.19 |
| Groundwater arsenic levels 3.0-9.9 μg/L (ppb) |  |  |  |  |  |  |
| No at risk | 19,931 | 14,287 | 9,751 | 3,546 |  |  |
| Cases/person-years | 183/153,878 | 186/182,261 | 177/183,965 | 25/39,403 |  |  |
| Adjusted HR (95% CI)^1^ | 1.00 | 0.99 (0.80, 1.23) | 1.03 (0.82, 1.29) | 0.78 (0.49, 1.25) | 0.96 (0.78, 1.17) | 0.68 |
| Groundwater arsenic levels ≥10.0 μg/L (ppb) |  |  |  |  |  |  |
| No at risk | 5,809 | 4,260 | 2,840 | 1,096 |  |  |
| Cases/person-years | 139/96,094 | 119/110,148 | 125/128,039 | 27/34,998 |  |  |
| Adjusted HR (95% CI)^1^ | 1.00 | 0.90 (0.69, 1.15) | 0.87 (0.67, 1.14) | 0.91 (0.55, 1.51) | 0.92 (0.73, 1.17) | 0.52 |

^1^ HRs (95% CIs) were estimated by Cox proportional hazard regression stratifying by age (years), gender (male or female), and cohorts (NHS, NHSII, or HPFS), and adjusting for ethnicity (Caucasian, Asian, African American and Hispanic/others), body mass index (<23.0, 23.0-24.9, 25.0-29.9, 30.0-34.9, or ≥35.0 kg/m^2^), smoking status [never smoked, past smoker, currently smoke (1-14, or ≥15 cigarettes/day)], physical activity (<3.0, 3.0-8.9, 9.0-17.9, 18.0-26.9, or ≥27.0 metabolic equivalent of tasks × hours/week), family history of myocardial infarction (yes or no), menopausal status and postmenopausal hormone use [pre-menopause, post-menopause (never, past, or current hormone use)], oral contraceptive use (never, past, or current use, for NHSII only), multivitamin use (yes or no), current aspirin use (yes or no), prevalent hypertension (yes or no), prevalent hypercholesterolemia (yes or no), prevalent diabetes (yes or no), total energy intake (kcal/day), and modified alternate Healthy Eating Index score (quintiles).

P value for interaction was 0.17 for coronary heart disease, and 0.48 for stroke.

HPFS, Health Professionals Follow-up Study; NHS, Nurses’ Health Study.

**Supplementary Table 5.** Results from sensitivity analyses, multivariable-adjusted hazard ratios (95% confidence intervals) of cardiovascular disease for ≥5 servings/week of rice consumption compared with <1 serving/week^1^

|  | Adjusting for individual dietary factors instead of modified aHEI score^2^ | Excluding participants who had history of hypertension at baseline | Updating dietary information every eight years instead of every four year |
| --- | --- | --- | --- |
| White rice | 0.95 (0.81, 1.10) | 0.93 (0.77, 1.13) | 1.02 (0.88, 1.19) |
| Brown rice | 0.98 (0.77, 1.24) | 0.85 (0.63, 1.16) | 0.97 (0.76, 1.24) |
| Total rice | 0.96 (0.87, 1.05) | 0.95 (0.85, 1.06) | 0.99 (0.90, 1.08) |

^1^ HRs (95% CIs) were estimated by Cox proportional hazard regression stratifying by age (years), gender (male or female), and cohorts (NHS, NHSII, or HPFS), and adjusting for ethnicity (Caucasian, Asian, African American and Hispanic/others), body mass index (<23.0, 23.0-24.9, 25.0-29.9, 30.0-34.9, or ≥35.0 kg/m^2^), smoking status [never smoked, past smoker, currently smoke (1-14, or ≥15 cigarettes/day)], physical activity (<3.0, 3.0-8.9, 9.0-17.9, 18.0-26.9, or ≥27.0 metabolic equivalent of tasks × hours/week), family history of myocardial infarction (yes or no), menopausal status and postmenopausal hormone use [pre-menopause, post-menopause (never, past, or current hormone use)], oral contraceptive use (never, past, or current use, for NHSII only), multivitamin use (yes or no), current aspirin use (yes or no), prevalent hypertension (yes or no), prevalent hypercholesterolemia (yes or no), prevalent diabetes (yes or no), total energy intake (kcal/day), and modified aHEI score (quintiles).

^2^ Adjusting for alcohol intake (0, 0.1-4.9, 5.0-9.9, 10.0-14.9, 15.0-29.9, or ≥30.0 g/day), poly-unsaturated-to-saturated fat ratio (quintiles), and intakes of *trans* fat, red meat, fish, fruits, vegetables, nuts, whole grain, coffee, and sugar-sweetened beverage (all quintiles) instead of modified aHEI score.

aHEI, alternate Healthy Eating Index; HPFS, Health Professionals Follow-up Study; NHS, Nurses’ Health Study.

**Supplementary Figure 1.** Multivariable-adjusted hazard ratios of cardiovascular disease according to combined categories of white rice consumption or brown rice consumption with body mass index (A, B), physical activity (C, D), smoking status (E, F), and modified alternate Healthy Eating Index score (G, H), respectively.

All HRs (95% CIs) were estimated by Cox proportional hazard regression stratifying by age (years), gender (male or female), and cohorts (NHS, NHSII, or HPFS), and adjusting for ethnicity (Caucasian, Asian, African American and Hispanic/others), body mass index (<23.0, 23.0-24.9, 25.0-29.9, 30.0-34.9, or ≥35.0 kg/m^2^), smoking status [never smoked, past smoker, currently smoke (1-14, or ≥15 cigarettes/day)], physical activity (<3.0, 3.0-8.9, 9.0-17.9, 18.0-26.9, or ≥27.0 MET-hours/week), family history of myocardial infarction (yes or no), menopausal status and postmenopausal hormone use [pre-menopause, post-menopause (never, past, or current hormone use)], oral contraceptive use (never, past, or current use, for NHSII only), multivitamin use (yes or no), current aspirin use (yes or no), prevalent hypertension (yes or no), prevalent hypercholesterolemia (yes or no), prevalent diabetes (yes or no), total energy intake (kcal/day), and modified alternate healthy eating index (quintiles).All bars meant 95% CI.

CVD, cardiovascular disease; HPFS, Health Professionals Follow-up Study; MET, metabolic equivalent of tasks; NHS, Nurses’ Health Study.


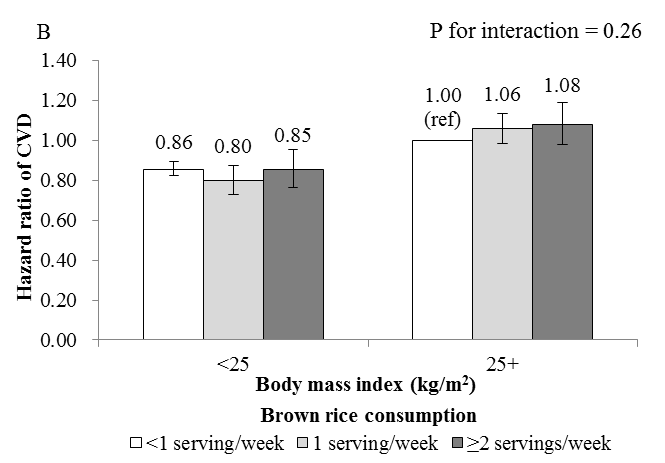

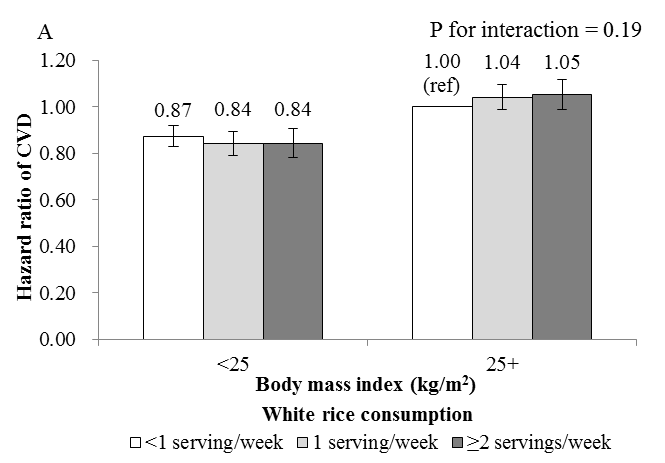


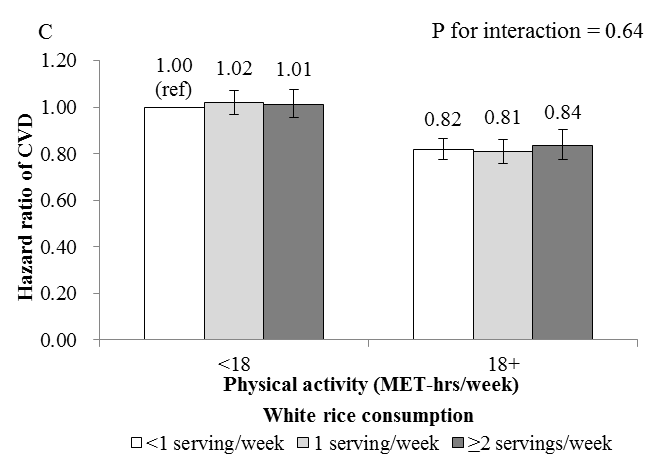
**
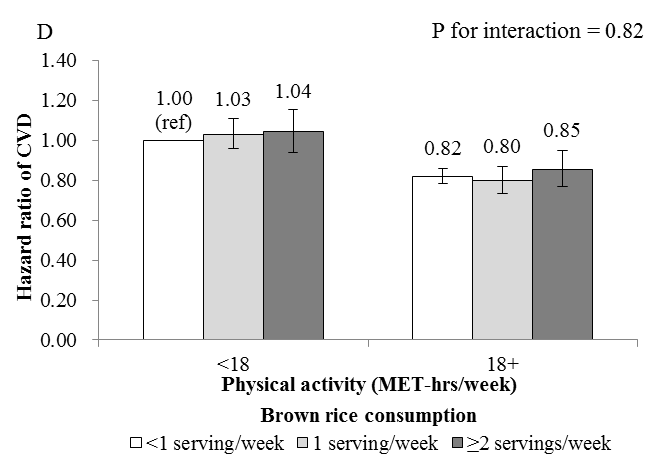

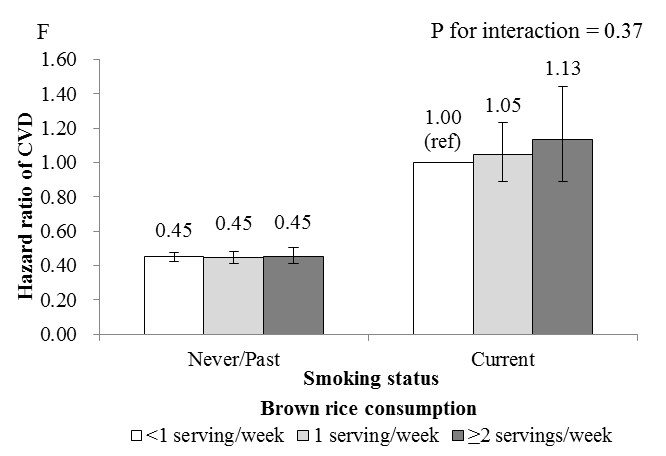
**
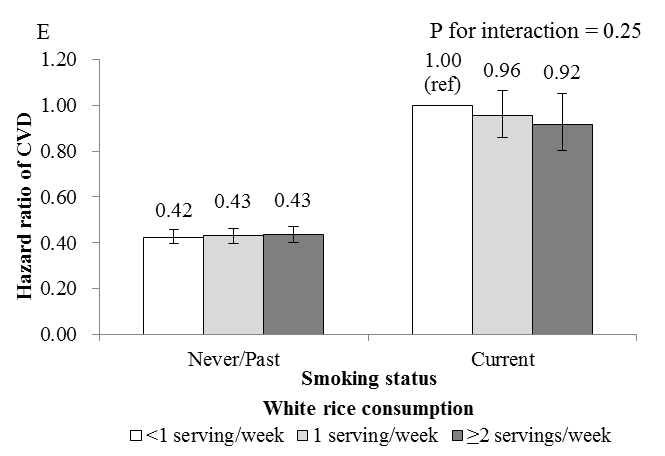
**Supplementary Figure 1.** (continued)

**Supplementary Figure 1.** (continued)


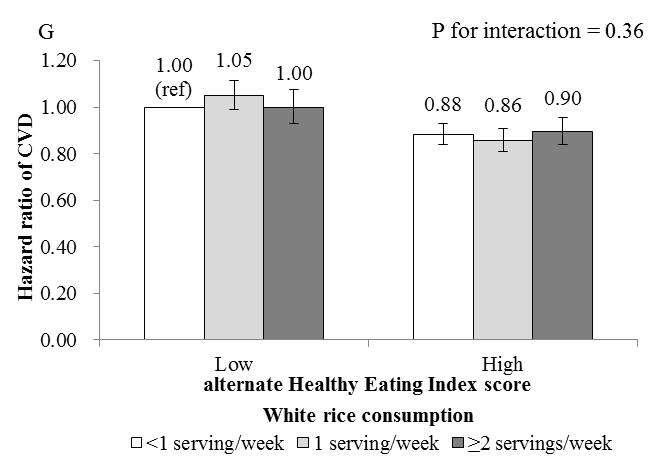

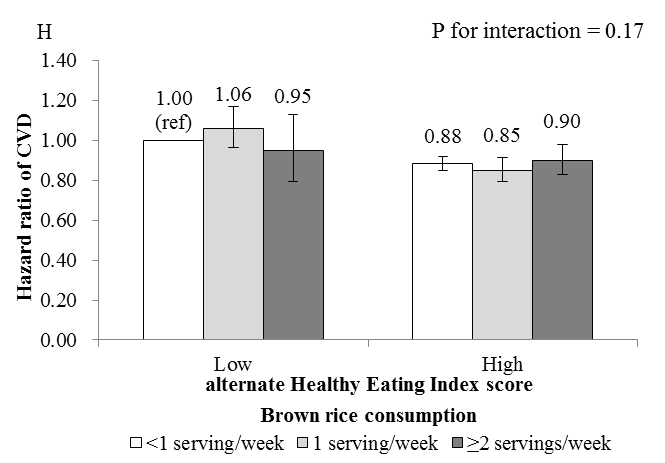


**References.**

1. Willett WC, Sampson L, Browne ML, Stampfer MJ, Rosner B, Hennekens CH, Speizer FE. The use of a self-administered questionnaire to assess diet four years in the past. Am J Epidemiol. 1988;127:188-199.

2. Rimm EB, Giovannucci EL, Stampfer MJ, Colditz GA, Litin LB, Willett WC. Reproducibility and validity of an expanded self-administered semiquantitative food frequency questionnaire among male health professionals. Am J Epidemiol. 1992;135:1114-1126.

3. Salvini S, Hunter DJ, Sampson L, Stampfer MJ, Colditz GA, Rosner B, Willett WC. Food-based validation of a dietary questionnaire: the effects of week-to-week variation in food consumption. Int J Epidemiol. 1989;18:858-867.

4. Feskanich D, Rimm EB, Giovannucci EL, Colditz GA, Stampfer MJ, Litin LB, Willett WC. Reproducibility and validity of food intake measurements from a semiquantitative food frequency questionnaire. J Am Diet Assoc. 1993;93:790-796.

5. U.S. Geological Survey. Trace Elements National Synthesis Project. Internet: http://water.usgs.gov/nawqa/trace/arsenic/ (Accessed November 25, 2013).

6. Rose G, Blackburn H. *Cardiovascular survey methods. (World Health Organization monograph series no. 56).* 2nd ed. Geneva, Switzerland: World Health Organization; 1982.

7. Walker AE, Robins M, Weinfeld FD. The National Survey of Stroke. Clinical findings. *Stroke.* 1981;12(2 Pt 2 Suppl 1):I13-I44.

8. Rich-Edwards JW, Corsano KA, Stampfer MJ. Test of the National Death Index and Equifax Nationwide Death Search. *Am J Epidemiol.* 1994;140(11):1016-1019.
